# Development and validation of a nomogram for predicting the probability of nontraumatic osteonecrosis of the femoral head in Chinese population

**DOI:** 10.1038/s41598-020-77693-9

**Published:** 2020-11-26

**Authors:** Qiang Xu, Hangjun Chen, Sihai Chen, Jing Shan, Guoming Xia, Zhiyou Cao, Xuqiang Liu, Min Dai

**Affiliations:** 1 Department of Orthopedics, The First Affiliated Hospital of Nanchang University, Artificial Joints Engineering and Technology Research Center of Jiangxi Province, Nanchang, 330006 Jiangxi province China; 2 Department of Gastroenterology, The First Affiliated Hospital of Nanchang University, Jiangxi Institute of Gastroenterology and Hepatology, Nanchang, 330006 Jiangxi province China

**Keywords:** Trauma, Diagnostic markers, Predictive markers

## Abstract

Although corticosteroids and alcohol are two major risk factors for nontraumatic osteonecrosis of the femoral head (NONFH), the effects of other factors have rarely been studied, thereby making early diagnosis and treatment of NONFH difficult. This study aimed to develop and validate a nomogram to NONFH, but patients with alcohol- and steroid-related NONFH are not at all taken into account in this study. A training cohort of 790 patients (n = 434, NONFH; n = 356, femoral neck fractures [non-NONFH]) diagnosed in our hospital from January 2011 to December 2016 was used for model development. A least absolute shrinkage and selection operator (lasso) regression model was used for date dimension reduction and optimal predictor selection. A predictive model was developed from univariate and multivariate logistic regression analyses. Performance characterisation of the resulting nomogram included calibration, discriminatory ability, and clinical usefulness. After internal validation, the nomogram was further evaluated in a separate cohort of 300 consecutive patients included between January 2017 and December 2018. The simple prediction nomogram included five predictors from univariate and multivariate analyses, including gender, total cholesterol levels, triglyceride levels, white blood cell count, and platelet count. Internal validation showed that the model had good discrimination [area under the receiver operating characteristic curve (AUC) = 0.80] and calibration. Good discrimination (AUC = 0.81) and calibration were preserved in the validation cohort. Decision curve analysis showed that the predictive nomogram was clinically useful. The simple diagnostic nomogram, which combines demographic data and laboratory blood test results, was able to quantify the probability of NONFH in cases of early screening and diagnosis.

## Introduction

Osteonecrosis of the femoral head (ONFH) is a pathological condition that is characterised by the necrosis of the bone marrow and osteocytes^1^ and can cause the gradual deterioration of the hip joint. A previous study reported that the incidence of ONFH could be as high as 20,000 cases per year in the United States^2^. In addition, Japan and South Korea report that there are an estimated 3,000 and 14,000 new cases each year, respectively^3^. In China, the total number of ONFH patients was estimated to be between 100,000 and 200,000 new cases^4^. Therefore, ONFH is attracting more attention as a health concern, and its aetiology and pathogenesis are constantly being investigated.

ONFH is mainly divided into two types: traumatic ONFH and nontraumatic ONFH (NONFH). NONFH has been reported to account for more than 70% of all ONFH cases^5^; thus, it is reasonable that NONFH is investigated further. The specific aetiological mechanism of NONFH has yet to be fully elucidated, which makes its early diagnosis and treatment difficult. Steroid^6^ and alcohol use^7^ have been identified as major risk factors for NONFH. Therefore, patients with these two factors require preventive action and prompt examination for early diagnosis. However, several studies have shown that NONFH is caused by multiple factors apart from steroid and alcohol use^8,9^. Moreover, NONFH caused by other factors is less likely to be diagnosed as patients show no symptoms in the early to middle stages. However, there have been few reports that have focused on identifying additional risk factors.

It has been hypothesised that NONFH is caused by vascular injury, bone cell physiological changes, oxidative stress, or insufficient blood supply^10–12^. Dyslipidaemia occurs in patients with NONFH, and there is a known relationship between decreased bone density and increased blood lipid in postmenopausal women^13^ and men^14^. Therefore, we hypothesised that dyslipidaemia may be a risk factor for NONFH. In addition, blood cells are closely related to many clinical diseases, and some of them are risk factors for NONFH^15^. Some studies have revealed a link between blood cell dysfunctions and clinical conditions, such as venous thrombosis and osteonecrosis in patients with sickle cell anaemia^16,17^. Studies have also shown that platelets or platelet-derived particles may cause a patient’s blood to be in a state of hypercoagulability, which may induce the formation of microthrombi in patients with NONFH^18^. Thus, haematological indicators are closely related to NONFH. From the above, previous studies that aimed to identify risk factors for NONFH have commonly focused on the correlation between abnormal blood lipids and abnormal haematological indicators for NONFH.

However, no model has been established to comprehensively evaluate risk factors or indicators that can aid with early diagnosis. Early diagnosis and intervention in patients with NONFH could effectively delay the progression of the disease or prevent the need for total hip arthroplasty, which is key to achieving good clinical results. Obviously, a reliable and effective diagnostic method is urgently needed to screen for NONFH and to achieve early diagnosis. Routine blood and biochemical tests are relatively easy to conduct through the detection of blood lipids and haematological indicators and can be analysed through traditional auxiliary diagnostic methods. Zeisler et al.^19^ used a laboratory test to predict clinically diseases. Furthermore, several combinations can improve detection by overcoming the low sensitivity and specificity of each individual test. In this study, we aimed to develop and validate a nomogram to estimate the probability of NONFH using clinical risk factors other than corticosteroids and alcohol consumption.

## Results

### Clinical characteristics

In the training cohort, 434 (55%) patients were diagnosed with NONFH, with an average age of 58.8 years; 356 (45%) patients were diagnosed with femoral neck fractures (non-NONFH), with an average age of 64.6 years. In the validation cohort, 150 (50%) patients were diagnosed with NONFH, with an average age of 58.68 years, while 150 patients were diagnosed with non-NONFH, with an average age of 64.81 years. Across the training and validation cohorts, 52.8% and 53.3% of patients with NONFH, respectively, were men. Despite the time differences, the baseline characteristics were comparable between the two cohorts, indicating that they were suitable for use as training and validation data sets. The demographic, laboratory, and clinical characteristics of the subjects in the NONFH and non-NONFH groups are summarised in Table 1. The variables in Table 1 were evaluated via univariate and multivariate logistic regression analyses. Significant differences between NONFH and non-NONFH groups were found with respect to gender, total cholesterol, triglyceride, WBC count, and platelet count. These variables were also identified (P < 0.05) as independent predictors of NONFH.

**Table 1:** Demographics and clinical characteristics of study subjects.

	Training cohort			Validation cohort		
	NONFH	Control	P	NONFH	Control	P
No. of patients, n (%)	434 (55)	356 (45)		150 (50)	150 (50)	
Age (Mean ± SD), years	58.8 ± 13.1	64.6 ± 18.1	< 0.001	58.68 ± 12.46	64.81 ± 18.04	< 0.001
**Gender, n (%)**						
Female	205 (47.2)	226 (63.5)	< 0.001	70 (46.7)	91 (60.7)	0.015
Male	229 (52.8)	130 (36.5)		80 (53.3)	59 (39.3)	
**Hypertension, n (%)**						
No	344 (79.3)	238 (66.9)	< 0.001	118 (78.7)	115 (76.7)	0.677
Yes	90 (20.7)	118 (33.1)		32 (21.3)	35 (23.3)	
**Diabetes, n (%)**						
No	404 (93.1)	313 (87.9)	0.013	142 (94.7)	130 (86.7)	0.017
Yes	30 (6.9)	43 (12.1)		8 (5.3)	20 (13.3)	
Total cholesterol (mmol/L)	4.44 ± 1.06	4.21 ± 0.91	< 0.001	4.59 ± 0.93	4.34 ± 0.96	0.025
Triglyceride (mmol/L)	1.10 (0.78–1.64)	0.87 (0.65–1.22)	< 0.001^U^	1.25 (0.82–1.74)	0.83 (0.61–1.11)	< 0.001^U^
HDL (mmol/L)	1.18 (0.99–1.45)	1.28 (1.06–1.51)	< 0.001^U^	1.29 ± 0.37	1.44 ± 0.39	< 0.001
LDL (mmol/L)	2.68 (2.14–3.24)	2.41 (1.93–2.87)	< 0.001^U^	2.80 ± 0.82	2.48 ± 0.80	< 0.001
White blood cell (10^9/L)	6.08 ± 1.97	7.69 ± 2.64	< 0.001	5.83 ± 1.56	7.40 ± 2.02	< 0.001
Red blood cell (10^12/L)	4.22 (3.91–4.64)	4.05 (3.65–4.43)	< 0.001 ^U^	4.31 ± 0.48	3.96 ± 0.62	< 0.001
Hemoglobin (g/L)	126.74 ± 51.65	119.72 ± 18.45	0.015	129.90 ± 14.99	120.38 ± 18.41	< 0.001
Platelet (10^9/L)	214.58 ± 77.67	175.99 ± 72.21	< 0.001	226.45 ± 68.75	202.09 ± 84.47	0.007

*SD* standard deviation, ^*U*^ Mann–Whitney U test, Median (interquartile range); *HDL* high-density lipoprotein, *LDL* low-density lipoprotein.

### Feature selection and independent risk factors for NONFH

**Figure 1:**
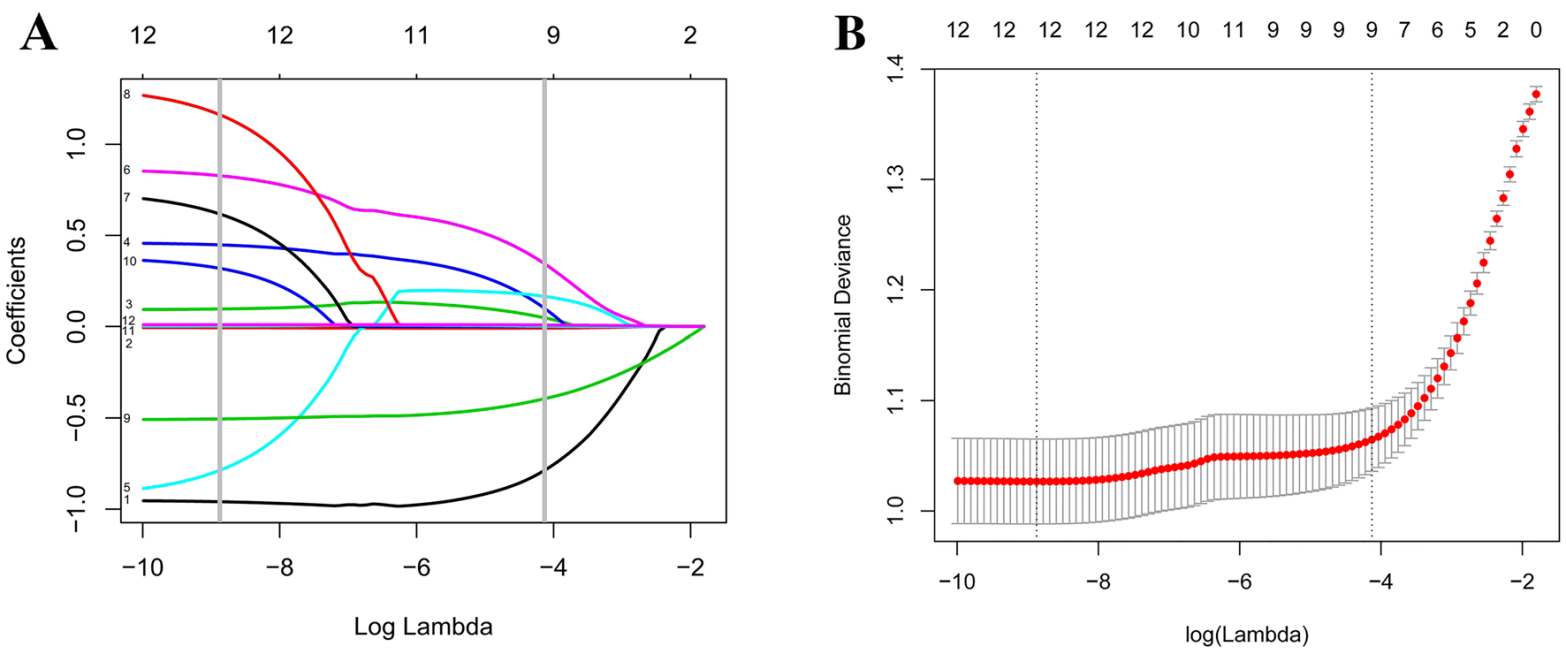
Predictor feature selection using the least absolute shrinkage and selection operator (lasso) logistic regression model. (**A**) The tuning parameter (λ) was determined in the lasso model by using a tenfold cross-validation and a minimum criterion. Notes: 1: Sex, 2: Age, 3: Hypertension, 4: Diabetes, 5: Total cholesterol, 6: Triglyceride, 7: High density lipoprotein, 8: low-density lipoprotein, 9: White blood cell, 10: Red blood cell, 11: Hemoglobin, 12: Platelet (**B**) To contrast the profile of the log (λ) sequence-generated coefficients, the lasso coefficient profiles of the 12 features resulted in 5 non-zero coefficients when using the optimal λ. The number at the top of the graph refers to the number of risk factor variables.

In texture features (Fig. 1A,B), based on the 790 patients in the training cohort, 12 features were simplified to 9 potential predictors that displayed non-zero coefficients in the lasso logistic regression model^20^. All predictive variables are listed in Table 2. Univariate analysis identified age, gender, hypertension, diabetes, total cholesterol, triglyceride, WBC count, haemoglobin, and platelet count as candidate risk factors for stepwise multivariate NONFH. Multivariate analysis showed that gender (odds ratio [OR] = 2.769, 95% confidence interval [CI] 1.876–4.088), total cholesterol, triglyceride, WBC count (OR = 0.603, CI % 0.546–0.666), and platelet count were independent risk factors for NONFH (Table 2). In addition, as shown in Supplementary Table 1, the intercept and betacoefficients/odds ratio’s of the multiple logistic regression analysis after dichotomy.

**Table 2:** Univariate and multivariate analysis in train cohort.

Variable	Univariate analysis		Multivariate analysis	
	OR (95% CI)	P value	OR (95% CI)	P value
Age	0.976 (0.966–0.985)	< 0.001	0.991 (0.979–1.003)	0.148
**Gender**				
Female	Reference		Reference	
Male	1.942 (1.458–2.586)	< 0.001	2.769 (1.876–4.088)	< 0.001
**Hypertension**				
No	Reference		Reference	
Yes	0.528 (0.383–0.727)	< 0.001	0.865 (0.571–1.310)	0.494
**Diabetes**				
No	Reference		Reference	
Yes	0.541 (0.331–0.881)	0.014	0.663 (0.357–1.230)	0.192
Total cholesterol	1.278 (1.104–1.479)	0.001	1.229 (1.005–1.503)	0.045
Triglyceride	2.031 (1.595–2.587)	< 0.001	1.941 (1.444–2.608)	< 0.001
White blood cell	0.721 (0.670–0.776)	< 0.001	0.603 (0.546–0.666)	< 0.001
Hemoglobin	1.013 (1.005–1.020)	< 0.001	1.009 (0.998–1.019)	0.098
Platelet	1.007 (1.005–1.010)	0.001	1.010 (1.008–1.013)	< 0.001

*HDL* high-density lipoprotein, *LDL* low-density lipoprotein.

### Diagnostic nomogram screening depending on the training cohort

**Figure 2:**
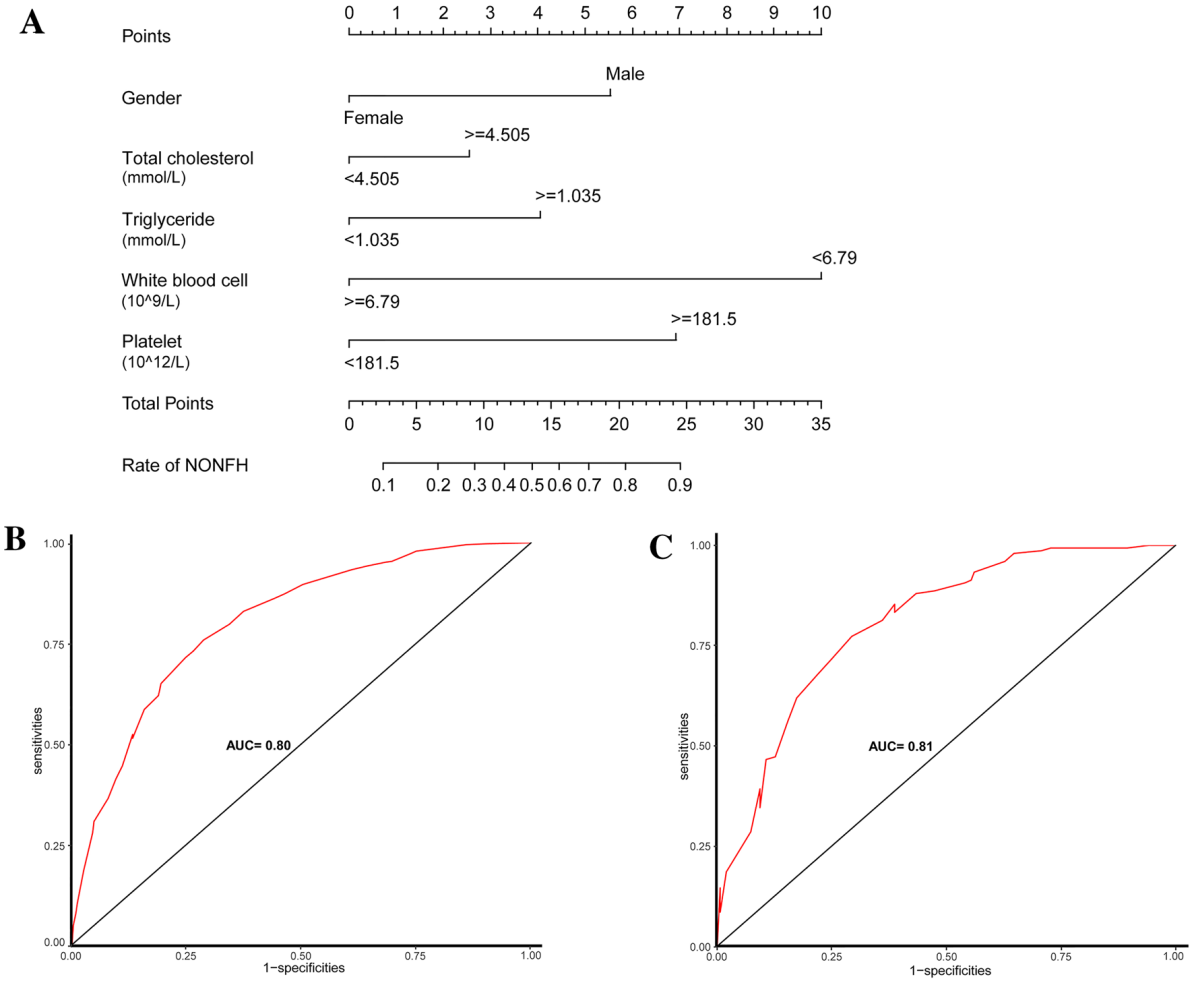
(**A**) The diagnostic nomogram for assessing NONFH. Gender, total cholesterol, triglyceride, white blood cell count, and platelet count were selected to be included in the nomogram. A receiver operating characteristic curve was used to evaluate the discriminatory ability of the model in the training (**B**) and validation (**C**) cohorts. The area under the curve (AUC) was 0.80 and 0.81, respectively, for each cohort.

A model containing these independent predictors was established and displayed as a nomogram (Fig. 2A). A nomogram containing five important diagnostic factors was presented. To use the nomogram, first, the gender of the subject was positioned on the relevant axis. Next, a straight line was drawn up to the top point axis to obtain the points based on gender. This process was then repeated for each covariate, and the total score was calculated by adding all points obtained from each covariate. The final summation laid on the total-points axis, and a straight line drawn down from there yielded the probability of NONFH.

### Validation of the diagnostic nomogram

**Figure 3:**
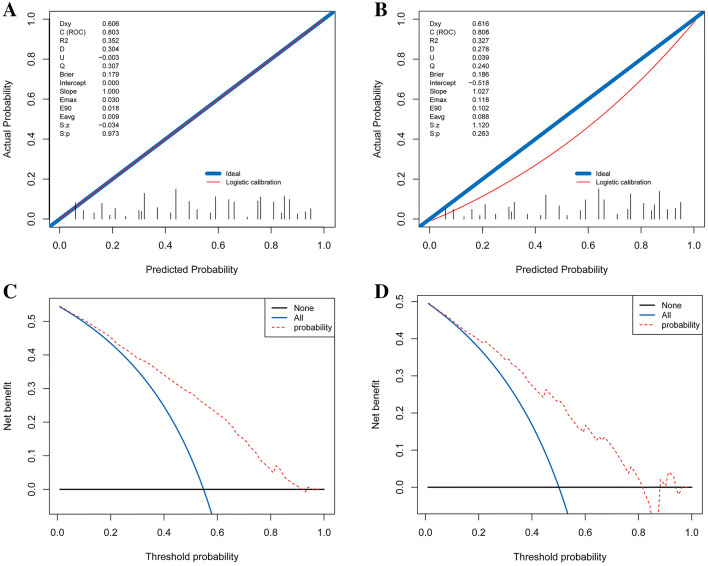
Calibration curves for the prediction models in the training (**A**) and validation (**B**) cohorts. These curves describe the calibration of the nomogram based on the consistency between the predicted risk and actual NONFH diagnosis. The x- and y-axis represent the predicted risks and actual result, respectively. The blue line represents the ideal prediction; the red line represents the performance of the nomogram. When the red line approaches the ideal prediction line, the prediction accuracy of the nomogram increases. The decision curve analysis of the nomogram and clinical models in the training (**C**) and validation (**D**) cohorts are presented. The x- and y-axis represent the threshold probability and net income, respectively. The red line represents the net benefit of the nomogram; the blue line assumes that all patients developed NONFH; and the black line assumes that no patients developed NONFH. The generated curve shows that using this model to identify patients who may develop NONFH is superior to a ‘treat-all-patients’ or ‘treat-no-patient’ management approach.

For the internal verification, the ROC showed that the resulting model had a fairly good discriminatory ability (Fig. 2B) with an AUC of 0.80 (0.77–0.83). The Hosmer–Lemeshow test showed no statistical significance (P = 0.973), the slope was 0, and the intercept was 1, indicated that the model fit well. In addition, the calibration plan was graphically displayed, and the predicted and observed data agreed well in the training cohort (Fig. 3A). In the independent validation cohort, the model demonstrated good discriminatory ability with an AUC of 0.81 (0.76–0.86) (Fig. 2C). As shown in the calibration curve (Fig. 3B), the non-statistical significance (P = 0.263) obtained in the Hosmer–Lemeshow test, the slope was 1.027, and the intercept was − 0.518, which also indicated good calibration.

### DCA curve analysis

Figure 3C,D show the DCA of the nomogram. The decision curve resulting from both the training cohort or validation cohort demonstrated that the capacity of the model to predict the occurrence of NONFH was superior to a ‘treat-all-patients’ or ‘treat-no-patient’ management approach. Overall, the nomogram was feasible and could be used to make reasonable predictions.

## Discussion

In this study, demographic, clinical, and laboratory data from several patients with NONFH were analysed to examine the association between putative risk factors, beside corticosteroids and alcohol, and NONFH. New predictive models, including lasso regression and a nomogram based on multiple logistic regression, were developed and validated for the diagnosis of NONFH. To the best of our knowledge, this is the first attempt to develop a diagnostic method for NONFH based on a nomogram. Our results provide a new perspective on relevant diagnostic criteria for NONFH and has strong translatability to clinical application.

Currently, the clinical diagnosis of NONFH is generally based on the symptoms and signs of the disease, as well as radiography and magnetic resonance imaging (MRI)^13^. However, obvious X-ray changes often indicate that the disease has reached the middle or late stages. Hip osteoarthritis caused by advanced NONFH may even cause the patient to lose the ability to work. Therefore, it is important to predict and diagnose NONFH early and prevent its advancement^21^. As the gold standard for NONFH diagnosis, MRI can provide relevant information for the early diagnosis of the disease. However, due to test interference and cost, it cannot be applied to NONFH screening. Zhao et al.^22^ reported that only 36.70% of NONFH cases were newly diagnosed after radiographic or MRI examination, and some of them were asymptomatic. Moreover, the majority of patients (86.25%) were in the early or middle stages of disease progression. Early and accurated diagnosis of NONFH using existing diagnostic methods is challenging. To improve clinical decision-making, patients and doctors need to know how often NONFH is diagnosed early. Notably, the present study integrated multiple independent risk factors for predicting NONFH. This approach could enhance clinical practice by establishing a more effective model for the diagnosing NONFH and potentially improve the capacity to distinguish between NONFH and non-NONFH.

In the present study, almost all categorical and continuous variables were significantly different between the NONFH and control groups. This difference may be attributed to the fact that femoral neck fractures occurred more in the elderly control group, while the NONFH group was relatively young. Older patients usually have underlying diseases, such as hypertension and diabetes, when compared to patients with NONFH.

Based on the large sample size, the current study provided the following major new findings: (1) gender was an independent predictor of NONFH; (2) triglyceride, total cholesterol, and platelet count were higher in the NONFH group than in the non-NONFH group, otherwise WBC count; and (3) when triglyceride was combined with other predictors (including gender, total cholesterol, platelet, and WBC count), the model’s discriminatory ability significantly improved. The higher prevalence of NONFH in men may be attributed to the confounding effects of alcohol, which is a major risk factor for NONFH. Irrespective of the levels of chronic abuse, typically men drink more than women. Overall, our results showed the nomogram to have acceptable performance, which is promising for its clinical application in the early diagnosis of NONFH.

Many studies have shown that lipid metabolism disorders may be important contributors to NONFH^22–24^. In a study by Zhang^25^ on 223 patients with NONFH showed that total cholesterol level was significantly higher than those in the control group. Other researchers have followed up on patients with femoral neck fractures and found that patients with elevated total cholesterol and triglyceride levels had a higher probability of NONFH than those with normal levels^23^. This finding suggests that total cholesterol and triglyceride levels are risk factors for NONFH. This is consistent with the results from our study where total cholesterol and triglyceride levels were higher in the NONFH group than in the control group. Furthermore, both parameters can be used as independent factors and diagnostic criteria for NONFH. Dyslipidaemia is still associated with NONFH. From the perspective of basic science, dyslipidaemia easily damaged vascular endothelial cells, reducing their ability to produce nitric oxide, which could lead to vasoconstrictive dysfunction and affect microcirculation. If it is carried out, it will cause fat emboli in the peripheral blood, which will block the blood vessels supplying the femoral head, and the blood supply will be interrupted, eventually resulting in NONFH.

WBCs, which are produced in the bone marrow, play important immune functions throughout their systemic circulation and are important in the body's defence against infection^26^. Yamaguchi et al.^27^ found that the level of pro-inflammatory cytokine, interleukin-6 (IL-6), increased in avascular necrosis of the femoral head. Some cytokines play an important role in the pathogenesis of femoral head necrosis. For example, in rats, the TLR4 signalling pathway, an important mediator of cytokine signalling, was shown to contribute towards lipopolysaccharide and methylprednisolone-induced necrosis of the femoral head^28^. Moreover, one study showed that plasma IL-33 may act as an alarm protein for NONFH, as a significant increase in its level may be related to disease progression^29^.

These studies suggest that leucocyte levels may play an important role in the pathogenesis of ONFH. Leucocytes can secrete various cytokines, such as interleukins, interferons, and tumour necrosis factor (TNF), which are involved in the regulation of immune response and inflammation. Inflammation is closely related to osteoporosis. IL-1, TNF-α, and IL-6, secreted by leucocytes, can induce a local aseptic inflammatory response while stimulating osteoblast expression and cause the release of a large amount of receptor activator of nuclear factor kappa B ligand. This release results in the excessive activation of osteoclasts and increased bone resorption, leading to early osteoporosis and microfracture of the femoral head and ultimately, osteonecrosis^30,31^. In addition, Daoussis et al. suggested that the Wnt pathway and IL-17 are novel regulators that are involved in local bone immune responses and bone remodelling processes^32^. Collectively, these studies indicate that the rate of NONFH occurrence is associated with an active immune defence and the ability for early leucocytes to clear necrotic tissues. The integrity of these processes may be associated with the inhibition of osteoclast formation and decreased bone resorption, thereby preventing femoral head necrosis. This interpretation is in line with our analysis, which reveals an association between leucocytes and NONFH. Under the stimulation of various factors, leucocytes can also regulate the activity of other proteases and cytokines and can promote the release of vascular endothelial growth factor (VEGF)^33,34^. VEGF plays a crucial role in promoting endothelial cell proliferation, angiogenesis, and revascularisation, which are important for preventing early femoral head necrosis^35^. Our research shows that WBC count was an independent predictor of NONFH.

Taal et al.^36^ aimed to identify factors that influenced bone mineral density and showed that NONFH was closely associated with haemoglobin levels. Mukisi-Mukaza et al.^17^ studied the relationship between NONFH, anaemia, and haemoglobin levels and showed that haemoglobin levels were positively correlated with NONFH. It is possible that higher haemoglobin concentrations resulted in higher blood viscosity. Collectively, these studies suggest a mechanism by which high haemoglobin levels contribute towards the development of NONFH. Our findings support that haemoglobin levels serve as a risk factor for the diagnosis of NONFH. However, in our analysis, the level of haemoglobin was not an independent risk factor. This could be due to the synergy between the level of haemoglobin and blood coagulation, which can cause thrombosis, thereby reducing the blood supply to the femoral head, and consequently leading to NONFH. Platelets are critically important blood cells and are mainly responsible for the body's coagulative function. Risk factors for NONFH include thrombosis due to coagulation dysfunction and vascular occlusion due to extravascular compression^37^. A study found that the thrombophilic mutation was commonly associated with and may be pathoetiologic for NONFH^38^. Coagulopathy plays a vital role in the pathogenesis of NONFH. Some studies suggest that platelet aggregation and activation are crucial in the pathogenesis of NONFH^39,40^. By contrast, other studies have found no significant difference in platelet counts between NONFH and non-NONFH groups^15,41^. Considering these different perspectives, our results show that platelet count is an independent risk factor for NONFH. This finding may provide important clues for future research on the aetiology of NONFH.

Although the current study provides useful information about the value of the nomogram for diagnosing NONFH, it has some limitations that must be acknowledged. Firstly, the nomogram was based on a single-centre retrospective study that could limit its applicability to other populations. Future external validation in independent datasets to confirm model robustness and generalizability in other populations is a prerequisite before the nomogram can be safely applied in clinical practice. Secondly, as the mechanism of NONFH is still unclear, a selection bias may exist. Due to the loss of some data in the retrospective study, weight/body mass index and smoking were not considered in the multiple regression analysis. The sensitivity and specificity of the nomogram could be further improved with multicentre retrospective validation studies or prospective randomised clinical trials, which will provide high-level evidence for future clinical applications.

## Conclusion

In conclusion, based on this large sample cohort study, we comprehensively evaluated the relationship between various conventional laboratory tests and NONFH. The successful development and careful evaluation of the NONFH diagnostic nomogram models, which include gender, total cholesterol, triglyceride, WBC count, and platelet count, provided satisfactory accuracy for predicting NONFH. This nomogram may be helpful for promoting the early diagnosis and prevention of NONFH.

## Patients and methods

### Human subjects and study design

**Figure 4:**
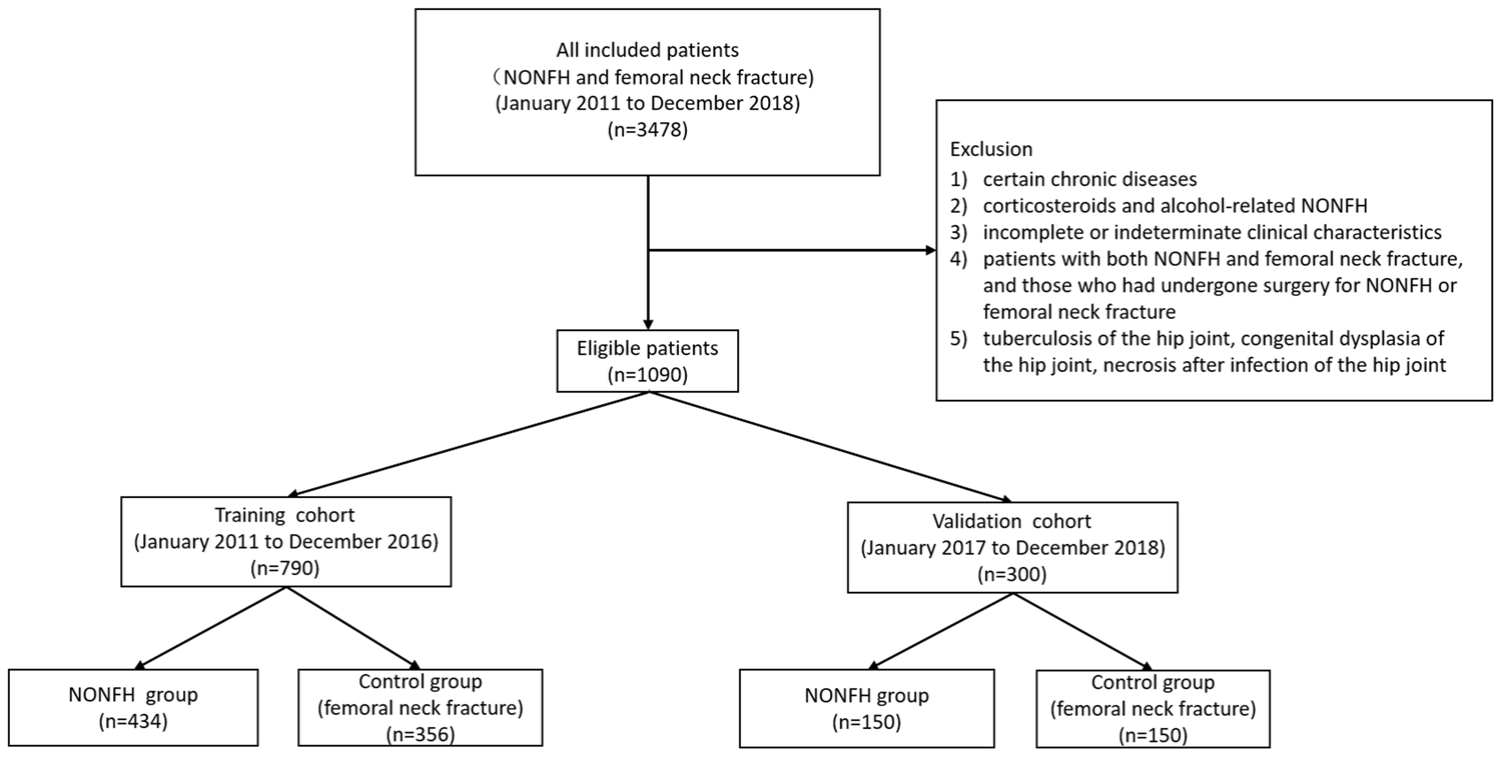
A flowchart describing patient selection and grouping. NONFH, nontraumatic osteonecrosis of the femoral head.

A total of 3478 patients were diagnosed with NONFH or femoral neck fractures in our institution from January 2011 to December 2018, and 1090 eligible patients were screened for this study (Fig. 4). From January 2011 to December 2016, 790 patients were included in the training cohort. In addition, a validation cohort of 300 patients was consecutively recruited, with the same criteria as those for the training cohort from January 2017 to December 2018. In the training cohort used to develop the nomogram, 434 patients in the experimental group were diagnosed with NONFH, and 356 patients in the control group were diagnosed with femoral neck fractures (non-NONFH). The exclusion criteria were as follows: (1) patients with certain chronic diseases (coronary heart disease, chronic kidney disease, chronic obstructive pulmonary disease, cancer); (2) incomplete or indeterminate clinical characteristics; (3) corticosteroid and alcohol-related NONFH (the patient had a medication history of prednisolone (or equivalent hormone) > 2 g for 3 months, with a corticosteroid history of more than 3 months, and was diagnosed as NONFH within 2 years; the average weekly drinking of pure alcohol > 320 g, with a drinking history of more than 6 months, and diagnosed as NONFH within 1 year); (4) patients with both NONFH and femoral neck fracture, and those who had undergone surgery for NONFH or femoral neck fracture; (5) tuberculosis of the hip joint, congenital dysplasia of the hip joint, and necrosis after infection of the hip joint. All methods were carried out in accordance with the relevant guidelines and regulations. This retrospective study was approved by the Ethics Committee of the first affiliated hospital of nanchang university. Informed consent was obtained from all subjects.

### Clinical information and assay of specific indicators for laboratory examination

The patient's age, gender, diagnosis, hypertension history, diabetes history, and other information were obtained from medical records. The patients underwent their first blood examination early in the morning on the day of admission. Commercial blood routine examination (SYSMEX XE-2100, SYSMEX CORPORATION Tokyo, Japan) and blood biochemical examination (automatic biochemical analyser, OLYMPUS 5421, Olympus Corporation, Tokyo, Japan) were used for detection and analysis. Total cholesterol, triglyceride, high-density lipoprotein, low-density lipoprotein, white blood cell (WBC) count, red blood cell count, haemoglobin level, and platelet count were measured.

### Statistical analyses

Continuous variables were tested for whether they followed a normal distribution using the Kolmogorov–Smirnov test. Normally distributed variables are presented with their mean ± standard deviation and were analysed using the independent t-test. Non-normally distributed variables are presented with their median (interquartile range) and were analysed using the Mann–Whitney U-test. Classification variables are expressed in frequency and percentage and were analysed using the Chi-squared test or Fisher exact test. Texture feature selection using the least absolute shrinkage and selection operator (lasso) regression model was employed for data dimensionality reduction and feature selection. Variables with P < 0.05 in univariate logistic regression were included in the multivariate model, where variables with P < 0.05 were considered as possible predictors^20^. Based on the multivariate analysis, a predictive nomogram was established in the training cohort. In order to evaluate the discriminative performance of the nomogram, the area under the receiver operating characteristic (ROC) curve (AUC) was measured. A calibration curve was generated for evaluating the calibration in combination with the Hosmer–Lemeshow test. Thereafter, the nomogram constructed in the training cohort was further verified in the validation cohort. The model performance for discrimination and calibration was evaluated in the validation cohort using the same methodology as described above. Finally, we assessed whether the model improved the predicted net income through a decision curve analysis (DCA). All tests were two-tailed, with a P-value of 0.05 being considered statistically significant. We used SPSS version 24 (IBM Corporation, Armonk, NY, USA) and R version 3.6.1 (R Foundation for Statistical Computing, Vienna, Austria; including the ‘rms’, ‘glmnet’, ‘ggplot2’, and ‘pROC’ functions) for data analyses.

## Supplementary information

Supplementary Information

## Data Availability

The datasets used and/or analyzed during the current study are available from the corresponding author on reasonable request.

## Abbreviations

ONFH: Osteonecrosis of the femoral head
NONFH: Nontraumatic osteonecrosis of the femoral head
LASSO: Least absolute shrinkage and selection operator
AUC: Area under the receiver operating characteristic curve
WBC: White blood cell
DCA: Decision curve analysis
MRI: Magnetic resonance imaging
