# Supplementary material for: Development and validation of a nomogram for predicting the probability of nontraumatic osteonecrosis of the femoral head in Chinese population
Source: Sci Rep. 2020 Nov 26;10:20660. doi: 10.1038/s41598-020-77693-9 (PMC7691506; doi:10.1038/s41598-020-77693-9)
Supplement: Supplementary file 1 — Supplementary Information [file 41598_2020_77693_MOESM1_ESM.docx]

| **Supplementary Table 1.** The intercept and the betacoefficients/odds ratio’s of the multiple logistic regression analysis after dichotomy. | | | |
| --- | --- | --- | --- |
| Variable | **β** | OR 95%CI | *P* value |
| Gender |  | | |
| Female | Reference |  |  |
| Male | 1.106 | 3.022(2.314-4.279) | 0.000 |
| Total cholesterol |  |  |  |
| <4.505 | Reference |  |  |
| ≥4.505 | 0.508 | 1.662(1.160-2.379) | 0.006 |
| Triglyceride |  |  |  |
| <1.305 | Reference |  |  |
| ≥1.305 | 0.808 | 2.244(1.587-3.175) | 0.000 |
| White blood cell |  |  |  |
| ≥6.79 | Reference |  |  |
| <6.79 | -1.996 | 0.136(0.094-0.196) | 0.000 |
| Platelet |  |  |  |
| <181.5 | Reference |  |  |
| ≥181.5 | 1.383 | 3.986(2.806-5.622) | 0.000 |
| Intercept | -0.709 | 0.492 | 0.000 |
